# Comparison of immunochemotherapy and chemotherapy alone in conversion therapy for locally advanced unresectable esophageal squamous cell carcinoma

**DOI:** 10.3389/fonc.2024.1370353

**Published:** 2024-06-24

**Authors:** Zhiyun Xu, Zhenbing You, Mengzhou Chen, Mingzhi Zhang, Cheng Shen, Dafu Xu, Keping Xu, Wenze Tian

**Affiliations:** Department of Thoracic Surgery, The Affiliated Huaian No.1 People’s Hospital of Nanjing Medical University, Huai'an, China

**Keywords:** esophageal cancer, conversion therapy, chemotherapy, immunotherapy, surgery

## Abstract

**Background:**

The clinical value of preoperative immunochemotherapy and simple chemotherapy induction regimen in the conversion therapy of locally advanced unresectable esophageal squamous cell carcinoma (ESCC) is still unclear.

**Method:**

Retrospective analysis was conducted on patients with unresectable cT_4b_ stage ESCC who underwent conversion surgery in our hospital from January 2020 to December 2022. According to the preoperative induction treatment plan, they were divided into induction immunochemotherapy group (iICT group) and induction chemotherapy group (iCT group). The conversion surgery rate, R0 resection rate, radiological and pathological tumor responses, safety, and short-term survival outcomes were analyzed.

**Results:**

The results showed that a total of 199 patients with cT_4b_ locally advanced unresectable ESCC who underwent preoperative induction therapy were included in this study. Among them, there were 64 cases (32.2%) in the iICT group, 135 cases (67.8%) in the iCT group. There was a statistically significant difference in objective response rate (73.5% vs 48.9%) and conversion surgery rate (81.3% vs 66.7%), between the iICT and iCT groups (*P*=0.001 and *P*=0.019). Among the two groups of patients who underwent surgery, there were statistically significant differences in R0 resection rate (94.2% vs 82.2%) and pathological complete remission rate (23.1% vs 6.7%) between the iICT and iCT groups (*P*=0.043 and *P*=0.004). And there was no statistically significant difference in the incidence of grade 3 and above between two groups (*P*=0.928). The 2-year EFS of the iICT group and iCT group were 76.4% and 42.4%, respectively, with statistically significant differences (*P*=0.006).

**Conclusions:**

Compared with simple chemotherapy, the combination of PD-1 inhibitors and chemotherapy can achieve better conversion surgery rate, tumor response and event-free survival in the conversion therapy of locally advanced unresectable ESCC.

## 1 Background

Esophageal carcinoma is one of the most common malignant tumors in the world. It is the 8th most common new malignant tumor and the 6th leading cause of death (1). In China, the 5-year survival rate for individuals with esophageal carcinoma is only approximately 20% (2). Preoperative neoadjuvant chemoradiotherapy or chemotherapy is the first choice for patients with locally advanced resectable esophageal squamous cell carcinoma (ESCC). These treatments can decrease tumor volume and postoperative recurrence, thereby improving surgical outcomes and patient prognosis (3, 4).

Due to the lack of serosa, ESCC easily penetrates the adventitia of the esophagus and invades adjacent organs (5). Based on Tumor-Node-Metastasis (TNM) staging, excluding distant metastasis, unresectable ESCC (cT_4b_ stage) is defined as tumor invasion of the aorta, trachea or bronchus and other organs and accounts for approximately 7% of all thoracic esophageal carcinomas (6, 7). The current standard of care for unresectable cT_4b_ ESCC is radical chemoradiotherapy or systemic chemotherapy alone (with contraindications to radiotherapy) (8). However, patients with cT_4b_ ESCC have a high recurrence rate and poor prognosis after radical chemoradiotherapy, with a 3-year survival rate of less than 30% (9). However, a small number of patients receive salvage surgery after recurrence, resulting in longer survival (10). In general, the treatment outcomes for patients with cT_4b_ ESCC are dismal, and therefore, new effective therapies or treatments are urgently needed.

Previous studies have found (11) that conversion therapy with preoperative induction chemotherapy or induction chemoradiotherapy can provide some cT_4b_ patients with locally advanced esophageal carcinoma the opportunity to undergo radical surgery and improve long-term survival. Recent studies have confirmed (4, 12), that immune checkpoint inhibitors, such as programmed death receptor-1 (PD-1) inhibitors, are widely used for the medical treatment of advanced esophageal carcinoma and neoadjuvant treatment of locally advanced esophageal carcinoma. The latest research reveals that chemotherapy combined with immunotherapy had a more favorable conversion effect for patients with unresectable ESCC (13–15). However, there are still relatively few research reports on the combination of PD-1 inhibitors and chemotherapy in the conversion therapy of ESCC.

In this study, the clinical data of patients with cT_4b_ locally advanced ESCC who underwent preoperative induction therapy and conversion surgery from February 2020 to December 2022 in the Department of Thoracic Surgery of our hospital were retrospectively analyzed. The aim of this study was to determine the clinical application value of two conversion therapy modes with preoperative immunochemotherapy or chemotherapy alone for patients with cT_4b_ stage advanced ESCC.

## 2 Materials and methods

### 2.1 Research objects

For patients initially diagnosed with unresectable ESCC, the standard treatment was curative chemoradiotherapy. Given the successful application of neoadjuvant therapy in resectable esophageal cancer patients. For some potentially resectable esophageal squamous cell carcinoma patients, such as those who invade the trachea or aorta, we will attempt a treatment strategy of preoperative induction and postoperative evaluation to determine the feasibility of curative surgery after sufficient communication with the patients.

Patients with unresectable locally advanced ESCC who received preoperative PD-1 inhibitors combined with chemotherapy or chemotherapy alone in our department from February 2020 to December 2022 were included this study. The inclusion criteria were as follows: ① patients between 18 and 75 years of age; ② preoperative pathological diagnosis of ESCC; ③ locally unresectable advanced stage esophageal carcinoma (cT4bN0–2M0), with extraversion of the trachea or aorta based on enhanced chest CT assessed by the multidisciplinary team (MDT) of our hospital. The exclusion criteria were as follows: ④ patients who received radiotherapy before surgery; ⑤ patients who received only one cycle of induction therapy before surgery; ⑥ patients who are expected to have poor cardiopulmonary function to receive surgical treatment. All patients will be followed up as of June 30, 2023. This study was approved by the hospital ethics committee (Ethics number KY-2023–042-01), and all patients provided informed consent and signed a consent form before treatment.

### 2.2 Preoperative conversion plan

**Figure 1:**
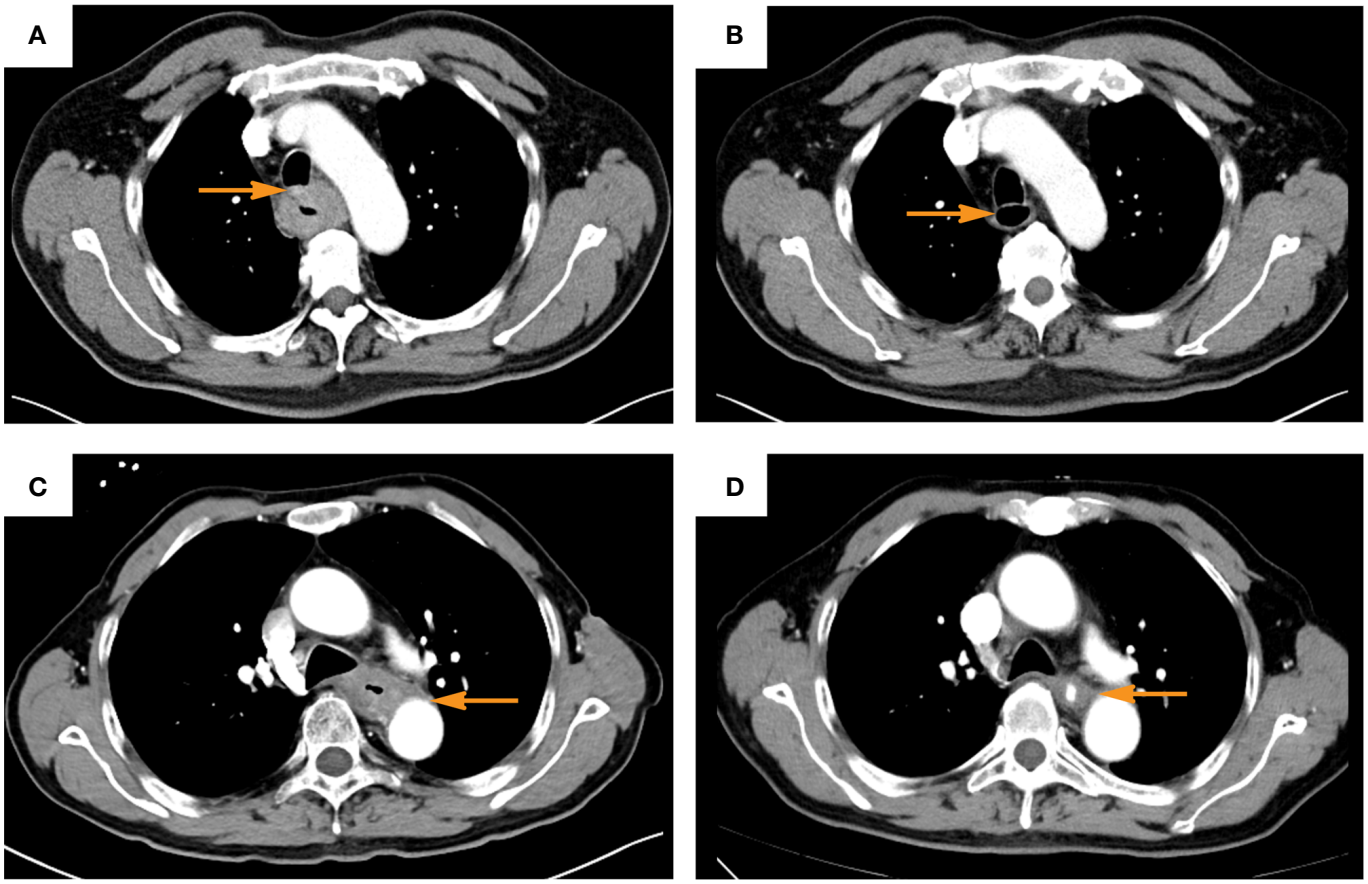
Typical computer tomography images of patients evaluated as clinic complete response after induction of immunochemotherapy **(A, C)**, before induction; **(B, D)** after induction; **(A, B)**, trachea invasion; **(C, D)**, aorta invasion. The yellow arrow indicates the area where the tumor invades the trachea or aorta before and after induction).

All the included patients received conversion therapy with immunochemotherapy or chemotherapy alone and two to three cycles of induction therapy (21 days between each cycle) before surgery. The amount of drugs used in conversion therapy was based on body surface area: for PD-1 inhibitors, camrelizumab or sintilimab 200 mg each time by intravenous infusion on the first day of each cycle; and for chemotherapy drugs, intravenous administration of albumin paclitaxel (260 mg/m^2^) on the 1st day and cisplatin (75 mg/m^2^) on the 1st-2nd days. During induction therapy, all patients were evaluated for conversion therapy efficacy via enhanced computer tomography (CT) of the chest and abdomen, and those deemed by the MDT team to have completely resectable tumors were treated with minimally invasive thoraco-laparoscopic esophagectomy (**Figure 1**). The presence of gaps between the tumor and the invading large blood vessels and trachea on CT imaging was used as an indicator for determining resectability. RECIST version 1.1 was used to evaluated the tumor response evaluation. For patients who are evaluated as inoperable or refuse to undergo surgery, synchronous radiochemotherapy (with a radiation dose of 60Gy) should be performed.

### 2.3 Follow-up

After the completion of conversion therapy, all patients were observed every 3 months, followed by CT scans of the neck, chest, and upper abdomen every 6 months thereafter. The diagnosis of recurrence is based on imaging. If necessary, 18 F-FDG-PET was used to confirm recurrence.

### 2.4 Observation indicators

Preoperative tumor regression and objective response rate (ORR) after conversion therapy were evaluated via CT, and induction drug-related adverse reactions, R0 resection rate, complete response (CR) rate, major pathologic response (MPR), tumor regression grading (TRG) (AJCC criteria), perioperative safety (postoperative hospital stay and postoperative complication rate) were analyzed.

### 2.5 Statistical processing

SPSS 18.0 was used for statistical processing. Measurement data that conformed to a normal distribution are expressed $\bar{x}$ ± s. The t test was used for intergroup comparisons, and the χ2 test was used to compare count data. *P*<0.05 was considered statistically significant. Kaplan-Meier curves were used to estimate event-free survival (EFS) from treatment date to relapse or death and to assess overall survival (OS). A two-sided P value of<0.05 was considered statistically significant.

## 3 Results

### 3.1 Baseline characteristics

A total of 199 patients with locally advanced unresectable cT_4b_ ESCC who underwent preoperative induction therapy were included in this study. Most patients were male (153, 76.9%) and average age was 64.5 years. Most patients were located in the middle esophagus, 13.6% of patients were located in the upper esophagus, and 16.6% of patients were located in the lower esophagus. The N stage was N_0_ in 124 patients (62.3%), N_1_ in 59 patients (29.6%), and N_2_ in 16 patients (8.0%). The patients were divided into an induction immunochemotherapy group (iICT) and a induction chemotherapy group (iCT) based the preoperative induction regimen. 47 patients (23.6%) received camrelizumab combined with chemotherapy before surgery, 17 patients (8.5%) received sintilimab combined with chemotherapy, and 135 patients (67.8%) received induction chemotherapy alone. Clinicopathological characteristics of the patients were summarized in **Table 1**.

**Table 1:** Clinicopathological characteristics of the patients.

Characteristics		All patients (n=199)	iICT (n=64)	iCT (n=135)	P value
Age, year		64.5 ± 6.12	64.7 ± 5.76	64.5 ± 6.30	0.775
Sex n(%)	Male	153 (76.9%)	49 (76.6%)	104 (77.0%)	0.941
	Female	46 (23.1%)	15 (23.4%)	31 (23.0%)	
History of smoking n(%)	Yes	90 (45.2%)	36 (56.3%)	62 (45.9%)	0.773
	No	109 (54.8%)	28 (43.8%)	73 (54.1%)	
Hypertension n(%)	Yes	49 (24.6%)	22 (34.4%)	27 (20.0%)	0.028
	No	150 (75.4%)	42 (65.6%)	108 (80.0%)	
Diabetes n(%)	Yes	17 (8.5%)	6 (9.4%)	11 (8.1%)	0.772
	No	182 (91.5%)	58 (90.6%)	124 (91.9%)	
Tumor location n(%)	upper portion	27 (13.6%)	5 (7.8%)	22 (16.3%)	0.166
	middle portion	139 (69.8%)	50 (78.1%)	89 (65.9%)	
	lower portion	33 (16.6%)	9 (14.1%)	24 (17.8%)	
cN n(%)	cN0	124 (62.3%)	45 (70.3%)	79 (58.5%)	0.276
	cN1	59 (29.6%)	15 (23.4%)	44 (32.6%)	
	cN2	16 (8.0%)	4 (6.3%)	12 (8.9%)	
BMI,Kg/m2		23.77 ± 3.21	23.88 ± 3.24	23.72 ± 3.21	0.747
Tumor extravasation	trachea	153 (76.9%)	53 (82.8%)	100 (74.1%)	0.172
	aorta	46 (23.1%)	11 (17.2%)	35 (25.9%)	
FEV1% n(%)	≥80%	162 (81.4%)	54 (84.4%)	108 (80.0%)	0.459
	<80%	37 (18.6%)	10 (15.6%)	27 (20.0%)	
Hemoglobin, g/L		121.2 ± 14.30	120.5 ± 15.59	121.6 ± 13.70	0.615
Albumin, g/L		44.0 ± 6.67	44.6 ± 8.09	43.7 ± 5.89	0.359
Induction cycle n(%)	2	109 (54.8%)	28 (43.8%)	74 (54.8%)	0.145
	3	90 (45.2%)	36 (56.2%)	61 (45.2%)	
Imaging response n(%)	CR	16 (8.0%)	12 (18.8%)	4 (3.0%)	0.000
	PR	97 (48.7%)	35 (54.7%)	62 (45.9%)	
	SD+PD	86 (43.3%)	17 (26.6%)	69 (51.1%)	
ORR (CR+PR) n(%)		113 (56.7%)	47 (73.5%)	66 (48.9%)	0.001

iICT, induction immunochemotherapy; iCT, induction chemotherapy; BMI, body mass index; cN clinic node stage; CR, complete response; PR, particial response; SD, stable disease; ORR, objective response rate.

### 3.2 Tumor regression

Sixteen patients (16 of 199, 8.0%) were assessed as clinical CR (cCR); 12 cases (12 of 64, 18.8%) were in the iICT group, and 4 cases (4 of 135, 3.0%) were in the iCT group, with a significant difference between the two groups (*P*< 0.001). 97 cases (97 of 199, 48.7%) were assessed as clinical PR (cPR); 35 cases (35 of 64, 54.7%) were in the iICT group, and 62 cases (62 of 135, 45.9%) were in the iCT group, with no significant difference between the two groups. The ORR was 73.5% in the iICT group and 48.9% in the iCT group, and the difference between the two groups was significant (*P*< 0.001) (**Table 1**).

The conversion surgery rate was 81.25% in the iICT group and 66.67% in the iCT group, and the difference between the two groups was significant (*P* =0.019). For the two groups of patients who underwent surgery, 12.7% (18/142) had pathological regression evaluated as pCR (TRG 0), accounting for 23.1% (12/52) of the iICT group and 6.7% (6/90) of the iCT group, respectively. The difference between the two groups was statistically significant (*P*=0.004). 29.6% (42/142) of the patients had pathological regression evaluated as MPR (TRG0+TRG1), with 48.1% (25/52) in the iICT group and 18.9% (17/90) in the iCT group. The difference between the two groups was statistically significant (*P<* 0.001) (**Table 2**).

**Table 2:** Radiological and pathological responses between induction chemotherapy and induction immunochemotherapy.

Characteristics		All patients (n=142)	iICT (n=52)	iCT (n=90)	P value
R0 n(%)	R0	123 (86.6)	49 (94.2)	74 (82.2)	0.043
	R1	19 (13.4)	3 (5.8)	16 (17.8)	
Postoperative hospital stay, d		14.2 ± 6.98	12.5 ± 3.28	15.2 ± 8.27	0.007
pT n(%)	pT0	18 (12.7)	12 (23.1)	6 (6.7)	0.004
	pT1	41 (28.9)	18 (34.5)	23 (25.6)	
	pT2	35 (24.6)	11 (21.2)	24 (26.7)	
	pT3	34 (23.9)	7 (13.5)	27 (30.0)	
	pT4	14 (9.9)	4 (7.7)	10 (11.1)	
pN n(%)	pN0	95 (66.9)	38 (73.1)	57 (63.3)	0.169
	pN1	37 (26.1)	13 (25.0)	24 (26. 7)	
	pN2	10 (7.0)	1 (1.9)	9 (10.0)	
TRG n(%)	0	18 (12.7)	12 (23.1)	6 (6.7)	0.003
	1	24 (16.9)	13 (25.0)	11 (12.2)	
	2	43 (30.3)	11 (21.1)	32 (35.6)	
	3	57 (40.1)	16 (30.8)	41 (45.5)	
Pathological response					
pCR n(%)	Yes	18 (12.7)	12 (23.1)	6 (6.7)	0.004
	No	124 (87.3)	40 (76.9)	84 (93.3)	
MPR n(%)	Yes	42 (29.6)	25 (48.1)	17 (18.9)	<0.001
	No	100 (70.4)	27 (51.9)	73 (81.1)	

iICT, induction immunochemotherapy; iCT, induction chemotherapy; pT, pathological tumor stage; pN, pathological nodal stage; TRG, tumor regression grade; pCR, pathological complete response; MPR, major pathological remission.

### 3.3 Safety comparison

Among the patients in the study, 110 (110 of 199, 53.3%) patients experienced at least one treatment-related adverse event (TRAE); 37 cases (37 of 64, 57.8%) were in the iICT group, and 73 cases (73 of 135, 54.1%) were in the iCT group, with no significant difference between the two groups (*P*=0.620). There were 4 patients (4 of 64, 6.3%) and 8 patients (8 of 135, 5.9%) with grade 3 TRAE or above in the iICT group and iCT group, respectively, and the difference between groups was no significant *(P*=0.928). Common TRAEs in the iICT group included reactive cutaneous capillary endothelial proliferation (RCCEP) (31.3%), vomiting (25%), leukopenia (23.1%), thrombocytopenia (21.9%) and pruritus (23.1%). Common TRAEs in the iCT group included vomiting (26.7%), leukopenia (23.7%), thrombocytopenia (14.8%) and anemia (11.9%) (**Table 3**).

**Table 3:** Adverse events during induction therapy.

Treatment-related adverse events	Grade, n (%)							
	iICT (n=64)				iCT (n=135)			
	Any	1	2	≥3	Any	1	2	≥3
Total	37 (57.8)	26 (40.6)	7 (10.9)	4 (6.3)	73 (54.1)	46 (34.1)	17 (12.6)	8 (5.9)
RCCEP	20 (31.3)	17 (26.6)	2 (3.1)	1 (1.6)	0 (0.0)	0 (0.0)	0 (0.0)	0 (0.0)
Vomit	16 (25.0)	11(17.2)	4 (6.2)	1 (1.6)	36 (26.7)	27 (20.0)	6 (4.4)	3 (2.2)
Liver dysfunction	3 (4.7)	2 (3.1)	1 (1.6)	0 (0.0)	9 (6.7)	7 (4.4)	2 (1.5)	0 (0.0)
Renal dysfunction	1 (1.6)	1 (1.6)	0 (0.0)	0 (0.0)	3 (3.3)	2 (1.5)	1 (0.7)	0 (0.0)
Thyroid dysfunction	3 (4.7)	3 (4.7)	0 (0.0)	0 (0.0)	0 (0.0)	0 (0.0)	0 (0.0)	0 (0.0)
Pruritus	12 (18.8)	8 (12.5)	3 (4.7)	1 (1.6)	4 (3.0)	3 (2.2)	1 (0.7)	0 (0.0)
Diarrhea	4 (6.2)	3 (4.7)	1 (1.6)	0 (0.0)	5 (3.7)	3 (2.2)	2 (1.5)	0 (0.0)
Thrombocytopenia	14 (21.9)	10 (15.6)	4 (6.2)	0 (0.0)	20 (14.8)	14 (10.4)	4 (3.0)	2 (1.5)
Neutropenia	15 (23.1)	10 (15.6)	4 (6.2)	1 (1.6)	32 (23.7)	21 (15.6)	8 (5.9)	2 (1.5)
Anemia	7 (10.9)	6 (9.4)	1 (1.6)	0 (0.0)	16 (11.9)	13 (9.6)	2 (1.5)	1 (0.7)

iICT, induction immunochemotherapy; iCT, induction chemotherapy; RCCEP, reactive cutaneous capillary endothelial proliferation.

The R0 resection rates for patients in the iICT group and iCT group were 94.2% (49 of 52) and 82.2% (74 of 90), respectively, and the difference was significant (*P*=0.043) (**Table 2**). Among the patients in the study, 24 (24 of 142, 16.9%) developed pulmonary infection after surgery; 8 patients (8 of 52, 15.4%) were in the iICT group, and 16 patients (16 of 90, 17.8%) were in the iCT group, with no significant difference between the two groups (*P*=0.714). Seven patients (7 of 142, 4.9%) developed anastomotic leakage after surgery; 2 patients (2 of 52, 3.8%) were in the iICT group, and 5 patients (5 of 90, 5.6%) were in the iCT group, with no significant difference between the two groups(*P*=0.650). In addition, one patient in each group died of a serious lung infection.

### 3.4 EFS and OS

**Figure 2:**
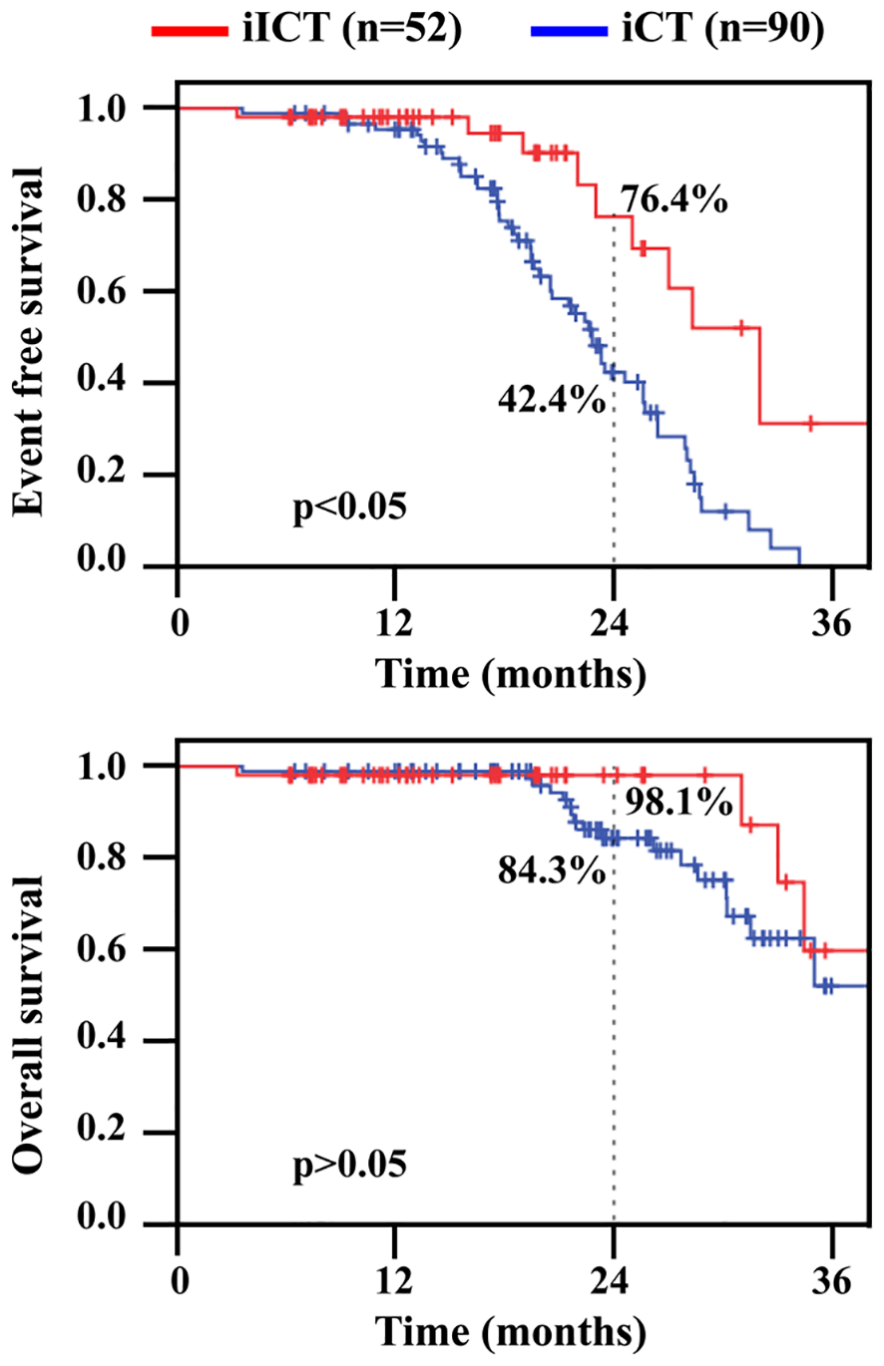
Event free survival (EFS) and overall survival (OS) in patients undergoing conversion surgery. iICT, induction immunochemotherapy; iCT, induction chemotherapy.

The median follow-up time for all patients was 21.5 months (IQR: 14.1–26.5). The 2-year EFS rates for patients in the iICT group and iCT group were 76.4% and 42.4%, respectively. There was a significant difference between the two groups (*P*=0.006). The 2-year OS rates for patients in the iICT group and iCT group were 98.1% and 84.3%, respectively. There was no significant difference in 2-year OS between the two groups (*P*=0.207) (**Figure 2**).

## 4 Discussion

Radical surgery after preoperative induction is becoming a new mode of treatment for cT_4b_ ESCC (9, 11). However, the application of an immunochemotherapy regimen in conversion surgery for cT_4b_ ESCC is still in its infancy. For the first time, we compared the effect of different conversion therapy with immunochemotherapy and chemotherapy alone for cT_4b_ ESCC. For patients undergoing conversion surgery, compared with chemotherapy alone, immunochemotherapy resulted in a higher conversion surgery rate, pCR rate, R0 resection rate and a better prognosis.

This study found that in patients with cT_4b_ ESCC, the induction regimen of preoperative PD-1 inhibitor combined with albumin-bound paclitaxel + cisplatin resulted in a better pCR rate than did the chemotherapy regimen of albumin-bound paclitaxel + cisplatin alone (23.1% vs. 6.7%). Our findings are consistent with the results reported by Huang (17) et al., which both confirm that immunochemotherapy results in a better pCR rate (22.4% vs. 6.7%) than does chemotherapy alone in the conversion treatment of cT_4b_ ESCC. The pCR rate after induction with preoperative chemotherapy in a previous study was 4% (9), which is similar to 6.7% for chemotherapy alone observed in this study. However, the pCR rate after induction by preoperative chemoradiotherapy was maintained at approximately 15% (13, 16–18). Fan (13) reported that the pCR rate was as high as 22.2% for patients undergoing conversion surgery after induction with preoperative immunochemotherapy. For patients with resectable locally advanced esophageal carcinoma who received neoadjuvant therapy, patients with a pCR had better long-term survival (19). Therefore, induction immunochemotherapy plus conversion surgery may benefit more patients because it yields better conversion surgery rate and pCR rate than preoperative chemotherapy or chemoradiotherapy.

A previous study (20) found that conversion surgery patients undergoing R0 radical resection had a high survival rate, comparable to that of cCR patients in a nonsurgical treatment group. The R0 resection rate is key to the success of conversion surgery in patients with locally advanced esophageal carcinoma. An retrospective study from Japan found that patients with cT4b ESCC after induction therapy with preoperative chemotherapy alone or chemoradiotherapy had R0 resection rates ranging from 32% to 100% (11, 18). In this study, the iICT group had a high R0 resection rate, i.e., 94.2%, which was significantly higher than that of the iCT group (82.2%). Fan (13) and Huang (17) also reported that conversion surgery after induction by immunochemotherapy yielded R0 resection rates as high as 81.5% and 94%, respectively.

The ultimate goal of conversion therapy for patients with locally advanced esophageal carcinoma is to improve long-term survival. Studies have confirmed the importance of R0 resection rate after conversion for improving prognosis (20). Previous studies have reported (11, 17) that the 3-year survival rate for patients with cT_4b_ ESCC after chemotherapy or chemoradiotherapy conversion surgery can increase to approximately 40% to 60%. Consistent with the results reported by Huang et al. (17), in this study, the 2-year EFS rate for patients receiving induction therapy combined with chemotherapy (76.4%) was significantly better than that for patients who received chemotherapy alone (42.4%). Although there was no significant difference in the 2-year overall survival rate between the two groups of patients in this study (98.1% vs. 84.3%), both were better than 31.5%, which was reported in the literature for patients receiving radical chemoradiotherapy (21). In general, the long-term survival of patients with cT_4b_ ESCC who received different conversion therapy regimens and successfully underwent surgery was mostly better than that of patients who received traditional chemoradiotherapy.

Drug safety during preoperative induction is closely related to treatment completion. Although the addition of immunotherapy increased the risk of RCCEP and thyroid dysfunction (12, 22), the addition of PD-1 inhibitors, compared with chemotherapy alone, did not increase overall grade ≥ 3 adverse events. Some studies have reported (13) that the esophageal mesentery may develop dense fibrosis after induction immunochemotherapy, leading to increased difficulty in surgery. Based on our experience, immunochemotherapy, with an ORR rate of 88.5%, significantly reduces the difficulty of surgery. In addition, there were no significant differences in the incidence of postoperative pneumonia and anastomotic leakage. Compared with those in the iCT group, postoperative pneumonia (15.4% vs. 18.9%), anastomotic leakage (3.8% vs. 5.6%) and other perioperative complications were not significantly increased in the iICT group.

The surgical conversion rate is also an important reference index for conversion therapy for locally advanced esophageal carcinoma. The surgical conversion rate of preoperative chemotherapy for cT_4b_ esophageal squamous cell carcinoma is 35%-85% (17); a recent prospective study (9) found that in patients with cT_4b_ ESCC after the induction of preoperative chemoradiotherapy, the surgical conversion rate was 81.82%. Fan (13) and Huang (17) reported that the conversion rate of immunochemotherapy in patients with cT_4b_ ESCC was 81.5% and 74.8%, respectively, and was not worse than the rate of conversion surgery after induction with radiotherapy and chemotherapy. Our study also confirms that the conversion surgery rate of immunotherapy combined with chemotherapy is 81.25%, which is significantly higher than 66.67% of chemotherapy alone. Future conversion surgeries also need to focus on the selection of induction protocols, surgical conversion rates, and how to early screen patients who are sensitive to induction therapy.

This study has several limitations. Firstly, this is a retrospective single-center study. Secondly, the sample size included in this study is relatively limited. Finally, the indications for surgical resection depend not only on the efficacy of conversion therapy, but also on the patient’s selection. Notwithstanding these research limitations, the advantages of our study encompass: (1) dependable data collection for each patient; (2) the follow-up duration is relatively protracted.

In general, based on the preliminary findings of this study, PD-1 inhibitors combined with albumin-bound paclitaxel + cisplatin, with a high conversion surgery rate, pCR rate, R0 resection rate and long-term survival rate, are more suitable for induction therapy before conversion therapy for cT_4b_ ESCC than is chemotherapy alone. The above results confirm that the induction regimen of preoperative immunochemotherapy has better application prospects for conversion therapy for patients with cT_4b_ ESCC. However, the exploration of conversion therapy for locally advanced unresectable ESCC has just started, and further large-scale, multicenter prospective research results are urgently needed to guide clinical practice. In the future, the exploration of conversion therapy for locally advanced ESCC should consider combinations of different conversion therapy methods, determine treatment cycles and treatment doses, and the effective control of adverse reactions and safety.

## Data availability statement

The raw data supporting the conclusions of this article will be made available by the authors, without undue reservation.

## Ethics statement

The studies involving humans were approved by Ethics Committee of The Affiliated Huaian No.1 People’s Hospital of Nanjing Medical University. The studies were conducted in accordance with the local legislation and institutional requirements. The participants provided their written informed consent to participate in this study.

## Author contributions

ZX: Writing – review & editing, Writing – original draft, Validation, Formal analysis, Data curation, Conceptualization. ZY: Writing – review & editing, Writing – original draft, Validation, Formal analysis, Data curation, Conceptualization. MC: Writing – review & editing, Writing – original draft, Validation, Formal analysis, Data curation, Conceptualization. MZ: Writing – review & editing, Resources, Methodology. CS: Writing – review & editing, Resources, Methodology. DX: Writing – review & editing, Resources, Methodology. KX: Writing – review & editing, Writing – original draft, Supervision, Resources, Project administration, Funding acquisition, Conceptualization. WT: Writing – review & editing, Writing – original draft, Supervision, Resources, Project administration, Funding acquisition, Conceptualization.
